# The risk of dyspnea in patients treated with third-generation P2Y_12_ inhibitors compared with clopidogrel: a meta-analysis of randomized controlled trials

**DOI:** 10.1186/s12872-020-01419-y

**Published:** 2020-03-17

**Authors:** Na Zhang, Weisen Xu, Ou Li, Bing Zhang

**Affiliations:** 1 Intensive Care Unit, China Emergency General Hospital, 29 Xibahenanli, Beijing, 100028 China; 2 Department of Stomatology, Beijing University of Chinese Medicine Third Affiliated Hospital, Beijing, 100029 China

**Keywords:** Ticagrelor, Prasugrel, Drug side effect, Meta-analysis

## Abstract

**Background:**

Ticagrelor and prasugrel are two third-generation oral P2Y_12_ inhibitors which are more commonly used in clinical practice. However, dyspnea has been consecutively reported in patients using third-generation oral P2Y_12_ inhibitors. This study aims to compare the risk of dyspnea in patients treated with third-generation P2Y_12_ inhibitors compared with clopidogrel.

**Methods:**

We systematically searched the PubMed, Cochrane Central Register of Controlled Trials databases, ClinicalTrials.gov and Web of Science for randomized control trials (RCTs) comparing ticagrelor or prasugrel with clopidogrel until July 2019. The primary outcome was the incidence of dyspnea. The risk ratios (RR) and 95% confidence intervals (CI) were estimated using meta-analysis.

**Results:**

We included 25 RCTs involving 63,484 patients in this meta-analysis, including 21 studies on ticagrelor and 4 studies on prasugrel. Compared to the clopidogrel group, third-generation oral P2Y_12_ inhibitors were associated with an increased risk of dyspnea compared with clopidogrel (RR 2.15, 95% CI 1.59–2.92), which was consistent in the analysis of ticagrelor (RR 2.65, 95% CI 1.87–3.76). However, the adverse effect was not found among patients receiving prasugrel therapy (RR 1.03, 95% CI 0.86–1.22). The increased dyspnea risk of ticagrelor was consistent in subgroups with different follow-up durations (≤ 1 month RR 1.87, 95% CI 1.56–2.24; 1–6 months RR 4.19, 95% CI 1.99–8.86; > 6 months 2.45, 95% CI 1.13–5.34).

**Conclusions:**

Ticagrelor has a higher risk of dyspnea than clopidogrel, which was not observed in patients using prasugrel.

## Introduction

Antiplatelet agents are the common primary therapy for patients with acute coronary syndrome (ACS), especially in patients undergoing percutaneous coronary intervention (PCI) [1]. Clopidogrel is the most commonly prescribed drug of antiplatelet agents. However, clopidogrel has a delayed onset and a modest antiplatelet effect. New antiplatelet agents, ticagrelor and prasugrel, were developed as third-generation oral P2Y_12_ inhibitors, which inhibit platelets more rapidly and persistently than clopidogrel. Several large randomized controlled trials (RCTs) have confirmed the superiority of third-generation P2Y_12_ inhibitors over clopidogrel in preventing ischemic vascular events [2–4].

Bleeding, as the most common side effects of third-generation oral P2Y_12_ inhibitors, has been well evaluated in previous studies [5–7]. Dyspnea was another important side effect, which was commonly reported in third-generation oral P2Y_12_ inhibitors users. The PLATO (Platelet Inhibition and Patient Outcomes) study showed that dyspnea was more common in the ticagrelor group than in the clopidogrel group (13.8% vs. 7.8%) [2]. More cases with dyspnea were also reported among patients taking prasugrel in the study of TRITON-TIMI-38 (Trial to Assess Improvement in Therapeutic Outcomes by Optimizing Platelet Inhibition with Prasugrel-Thrombolysis in Myocardial Infarction) [4]. However, the risk of dyspnea was not well established in previous studies on third-generation oral P2Y_12_ inhibitors, which mainly focused on their efficacy or the risk of bleeding. Therefore, we performed this meta-analysis of RCTs to compare the risk of dyspnea in patients taking third-generation oral P2Y_12_ inhibitors with clopidogrel.

## Methods

This meta-analysis was carried out according to the methods recommended in the Preferred Reporting Items for Systematic Reviews and Meta-Analyses (PRISMA) guidelines [8].

### Search strategy

RCTs comparing the safety of ticagrelor or prasugrel with clopidogrel published before July 2019 were identified from PubMed, Cochrane Central Register of Controlled Trials databases, ClinicalTrials.gov and Web of Science. The following terms, dyspnea or dyspnoea, and prasugrel or CS-747 or 640,315 or ticagrelor or AZD6140, and randomized controlled trial or random* were used.

### Inclusion and exclusion criteria

Two independent reviewers reviewed the eligible studies. Disagreements were resolved by discussion with a third reviewer. The inclusion criteria were as follows: 1) full-text RCTs; 2) comparing ticagrelor or prasugrel with clopidogrel; 3) dyspnea was reported as one of the safety endpoints; 4) in English. The exclusion criteria were as follows: 1) incomplete data; 2) reanalysis or subgroup analysis of previous RCTs; 3) including healthy subjects only or involving animals.

### Endpoints of evaluation

The primary outcome was the risk of dyspnea in patients taking third-generation oral P2Y_12_ inhibitors compared with clopidogrel. Dyspnea was reported by the participants and judged by the investigators. However, most studies did not specify the definition of dyspnea.

### Data extraction and risk of bias analysis

Characteristics and data of included studies were extracted by two independent reviewers, and disagreements were settled by discussion with a third reviewer. The extracted data included the year of publication, the study country or area, sample size, indications for treatment, dose of drugs, the duration of treatment, the duration of follow-up, and frequency of dyspnea in each study arm. Results of included studies were also checked in ClinicalTrials.gov. All randomized patients were included in this meta-analysis. Specifically, if the number of patients who received at least one dose of study drugs was specified in the included studies, this number would be used alternatively. If the data were incomplete, authors would be contacted for more information. The Cochrane Collaboration’s tool was used to assess the risk of bias of the studies [9]. Random sequence generation, allocation concealment, blinding, incomplete outcome data, and selective outcome reporting were assessed.

### Statistical methods

Pooled risk ratios (RRs) and corresponding 95% confidence intervals (CIs) were calculated in order to evaluate the risk of dyspnea for the third-generation oral P2Y_12_ inhibitors, in which random effects models were applied. For sensitivity analysis, we also computed the estimates using fixed effects model. I^2^ were taken as the determinant of heterogeneity and *P* value (< 0.1) indicated statistically significant. We regarded I^2^ values of < 25%, 25–50%, and > 50% as evidence of low, moderate, and high levels of heterogeneity, respectively [10]. Publication bias was assessed by using funnel plots. Begg’s rank correlation test and the Egger’s linear regression test were performed to test the symmetry of funnel plot [11, 12].

Furthermore, we also performed subgroup analyses on individual drug (ticagrelor or prasugrel), studies with standard dosage of drugs (maintenance dose of ticagrelor 90 mg twice per day, prasugrel 10 mg once per day and clopidogrel 75 mg once per day), studies involving Asian subjects, and studies according to study follow-up (≤ 1 month, 1–6 months, > 6 months). In addition, sensitivity analysis was also performed after excluding studies with high risk of bias or excluding the study with the largest sample size. R software, version 3.5.1 (R Foundation for Statistical Computing, Vienna, Austria, 2018) was used to perform this meta-analysis.

## Results

### Study characteristics and study quality

**Fig. 1:**
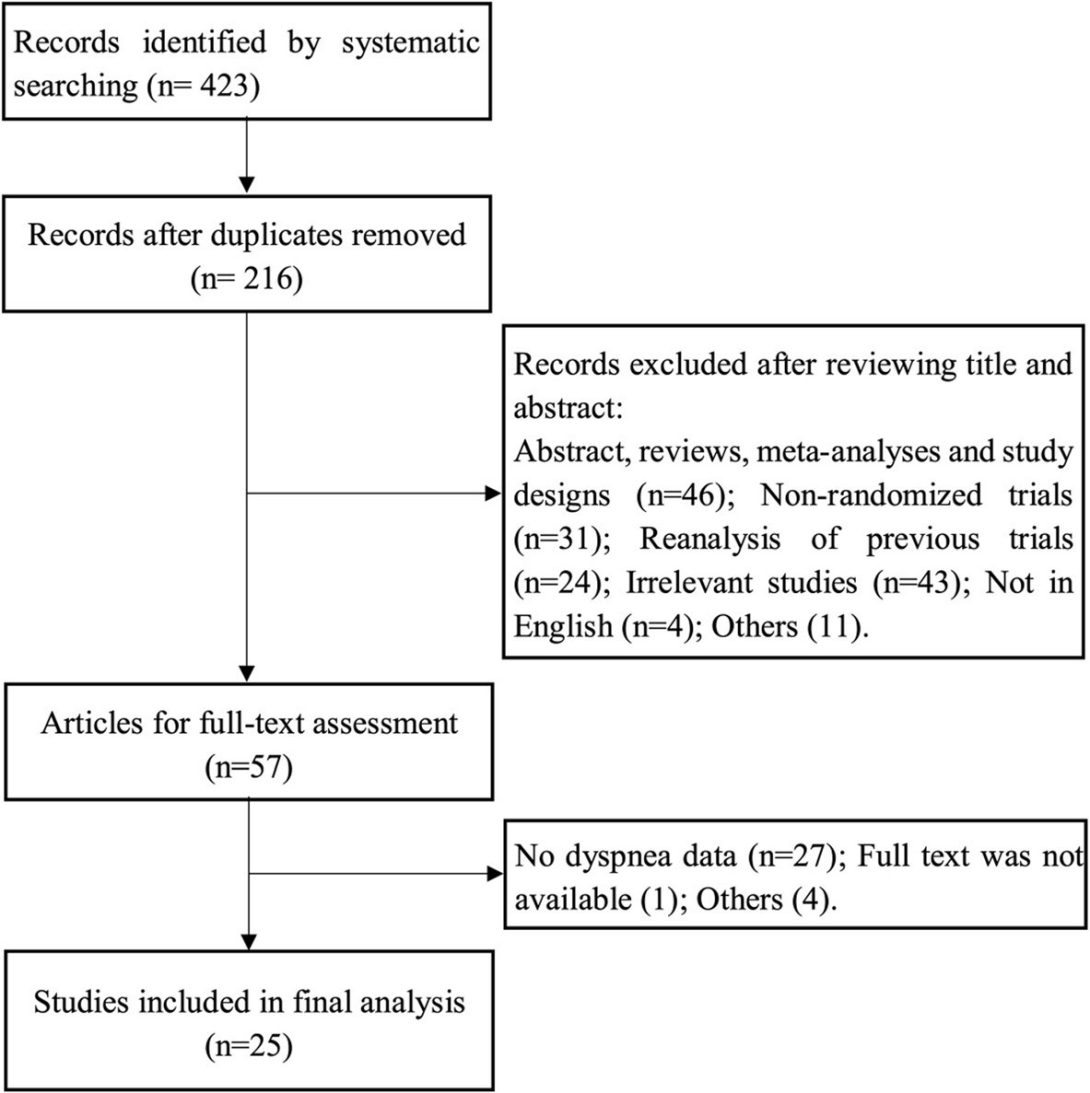
Flow diagram of the study selection

The study selection process is outlined in Fig. 1. After removing the duplicates, 216 relevant citations were identified, which yielded 25 studies fulfilling the inclusion criteria, including 21 studies comparing ticagrelor with clopidogrel [2, 3, 13–31] and 4 studies comparing prasugrel with clopidogrel [4, 32–34]. For study of Ge 2010 [32], the data was from ClinicalTrials.gov. A total of 64,049 patients were involved in the randomization, and 63,484 patients who received at least one dose of study drugs were included in the final analysis. The characteristics of included studies were summarized in Table 1. There were 10 ticagrelor studies [17–23, 25, 27, 31] and 1 prasugrel study [32] carried out in Asian population. Considering the dosage of study drugs, standard maintenance dose was used in 12 ticagrelor studies [2, 3, 15–17, 20, 21, 24–26, 28, 29] and 2 prasugrel studies [4, 34].

**Table 1:** Characteristics of included studies

Study (year)	Country or area	Indications	Randomized subjects^a^	Range of age	Third-generation P2Y_12_ inhibitors	Third-generation P2Y_12_ inhibitors, LD (mg, once) /MD (mg, BID or QD^b^)	Clopidogrel, LD (mg, once) /MD (mg, QD)	Duration of treatment	Duration of follow-up
Husted (2006) [13]	Denmark, Hungary and Norway	CAD	201	25–85	Ticagrelor	−/50–400	−/75	28 d	28 d
DISPERSE-2 (2007) [14]	14 countries	NSTEMI	990 (984)	≥18	Ticagrelor	−/90–180	300/75	4–12 w	12 w
PLATO (2009) [2]	43 countries	ACS	18,624 (18,421)	≥18	Ticagrelor	180/90	300–600/75	12 m	12 m
Onset/offset (2009) [15]	USA, UK	CAD	111	≥18	Ticagrelor	180/90	600/75	6 w	52 d
Bonello (2014) [16]	France	NSTEMI	60	–	Ticagrelor	180/90	600/75	–	1 m
PHILO (2015) [17]	Japan, South Korea and Taiwan	ACS	801 (767)	–	Ticagrelor	180/90	600/75	6–12 m	12 m
Li (2015) [18]	China	AMI or coronary artery in-stent restenosis with HTPR	48	20–80	Ticagrelor	180/90	−/150	–	6 m
Zhang (2016) [23]	China	ACS undergoing PCI	181	≥18	Ticagrelor	180/90	600/75–150	6 m	6 m
Xue (2016) [22]	China	NSTEMI	75	18–75	Ticagrelor	90–180/45–90	300/75	5 d	5 d
He (2016) [19]§	China	CAD	30	18–75	Ticagrelor	−/22.5	−/75	7 d	21 d
Lu (2016) [20]	China	ACS undergoing PCI	203	–	Ticagrelor	180/90	600/75	12 m	12 m
Wang (2016) [21]	China	STEMI with dementia	174	60–80	Ticagrelor	180/90	600/75	30 d	
Gu (2017) [27]	China	NSTEMI	76	–	Ticagrelor	180/90	600/150	–	3 m
Choi (2017) [25]	South Korea	DAPT after PCI	69	–	Ticagrelor	−/90¶	−/75	28 d	28 d
Dehghani (2017) [26]	Canada	STEMI undergoing PCI	144	≥18	Ticagrelor	180/90	300/75	–	30 d
EUCLID (2017) [3]	28 countries	Peripheral artery disease	13,885 (13,842)	≥50	Ticagrelor	−/90	−/75	–	30 d
Zafar (2017) [28]^c^	USA	CVD with T2DM	20	–	Ticagrelor	180/90	600/75	1 w	3 w
Campo (2017) [24]	Italy	CAD with COPD	46	≥18	Ticagrelor	180/90	600/75	6 m	1 m
TREAT (2018) [29]	10 countries	STEMI	3799 (3788)	≤75	Ticagrelor	180/90	300–600/75	–	30 d
Orme (2018) [30]	UK	CAD	180	≥18	Ticagrelor	180/60–90	600/75	30 d	30 d
Wu (2018) [31]	China	AMI undergoing PCI	257	–	Ticagrelor	180/60–90	300/75	–	1 y
TRITON-TIMI 38 (2007) [4]	30 countries	ACS	13,608 (13,457)	≥18	Prasugrel	60/10	300/75	6-15 m	6–15 m
Ge (2010) [32]	4 countries or areas	ACS	720 (692)	≥18	Prasugrel	30–60/5–10	300/75	90 d	90 d
TRILOGY-ACS (2012) [33]	52 countries	Unstable angina or NSTEMI without revascularization	9326 (9240)	≥18	Prasugrel	30/5–10	300/75	6–30 m	6–30 m
TRIGGER-PCI (2012) [34]	Germany and USA	CAD undergoing PCI with HTPR	423 (420)	18–80	Prasugrel	60/10	600/75	–	3–6 m

*ACS* acute coronary syndromes, *AMI* acute myocardial infarction, *BID* twice per day, *CAD* coronary artery disease, *COPD* chronic obstructive pulmonary disease, *d* days, *DAPT* dual antiplatelet therapy, *HPR* high platelet reactivity, *HTPR* high on-treatment platelet reactivity, *ITT* intention-to-treat, *LD* loading dose, *m* months, *MD* maintenance does, *NSTEMI* non-ST-elevated myocardial infarction, *PCI* percutaneous coronary intervention, *QD* once per day, *STEMI* ST-elevated myocardial infarction, *w* weeks

^a^ The number in the brackets is number of subjects that received at least 1 dose of the assigned study medication; ^b^ BID for ticagrelor, QD for prasugrel; ^c^ Only the first phase of the crossover study was included; ¶ Switched from clopidogrel

The quality assessment of the included studies is displayed in Table S1 and Figure S1. High risk bias was observed in some trials. As several studies were open-label trials [16, 23–25, 28, 30], performance bias and detection bias would be high. Though studies of Dehghani 2017 [26] and TREAT 2018 [29] were also open-label, the clinical endpoint assessment was blinded. In most studies, however, generation of random sequence and allocation concealment were not reported. Other biases were low in most studies.

### Dyspnea risk of third-generation P2Y_12_ inhibitors

**Fig. 2:**
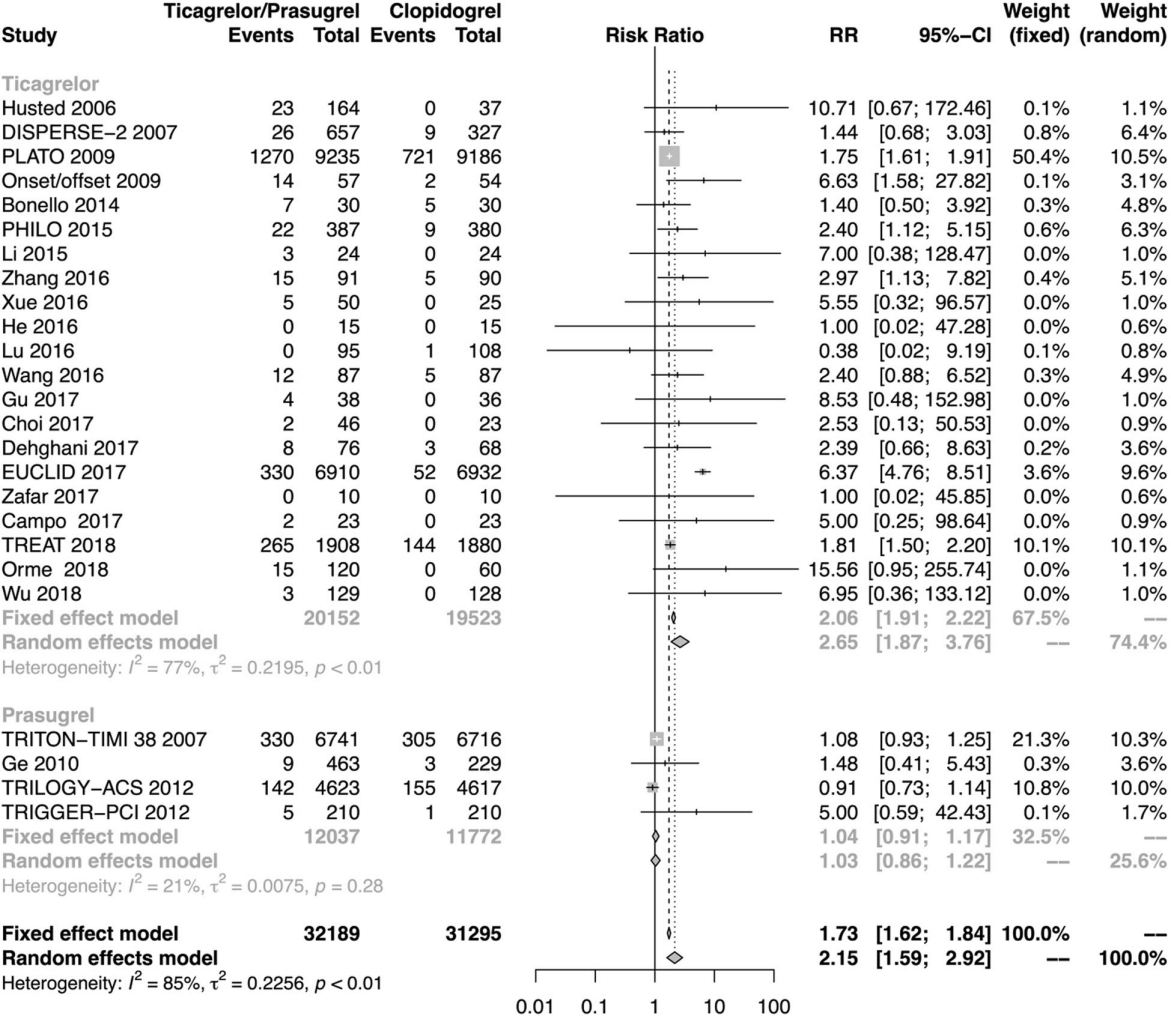
Forest plot of risk ratios for the incidence of dyspnea of third-generation P2Y_12_ inhibitors vs clopidogrel

All of the 25 studies were included in the analysis on dyspnea, involving a total of 63,484 patients (ticagrelor 20,152 vs clopidogrel 19,523; prasugrel 12,037 vs clopidogrel 11,772). In the included studies, 2512 (7.8%) cases of dyspnea were reported in the third-generation P2Y_12_ inhibitors group, and 1420 (4.5%) in clopidogrel group. Overall, third-generation P2Y_12_ inhibitors was associated with a higher risk of dyspnea compared with clopidogrel (RR 2.15, 95% CI 1.59–2.92, See Fig. 2). However, high heterogeneity was observed in this analysis with the I^2^ of 85% (*P* < 0.01). The result was consistent in subgroup analysis of ticagrelor (RR 2.65, 95% CI 1.87–3.76), but not in analysis of prasugrel (RR 1.03, 95% CI 0.86–1.22).

In addition, in subgroup analysis of studies with standard dose of drugs, the result was consistent (overall, RR 2.25, 95% CI 1.56–3.24; ticagrelor, RR 2.51, 95% CI 1.66–3.79; prasugrel, RR 1.58, 95% CI 0.43–5.80). Similar result was also observed in analysis of Asian studies (overall, RR 2.25, 95% CI 1.56–3.24; ticagrelor, RR 2.51, 95% CI 1.66–3.79; prasugrel, RR 1.58, 95% CI 0.43–5.80).

In the sensitivity analysis excluding studies with high risk of bias, 10 ticagrelor studies and all 4 prasugrel studies were included. The results remained consistent that third-generation P2Y_12_ inhibitors increased the risk of dyspnea (RR 2.22 95% CI 1.49–3.32), and it was also only observed among ticagrelor studies (RR 3.27, 95% CI 1.75–6.14). After excluding the largest study, the PLATO study, ticagrelor was still associated with increased risk of dyspnea (RR 2.90, 95% CI 1.85–4.55).

**Fig. 3:**
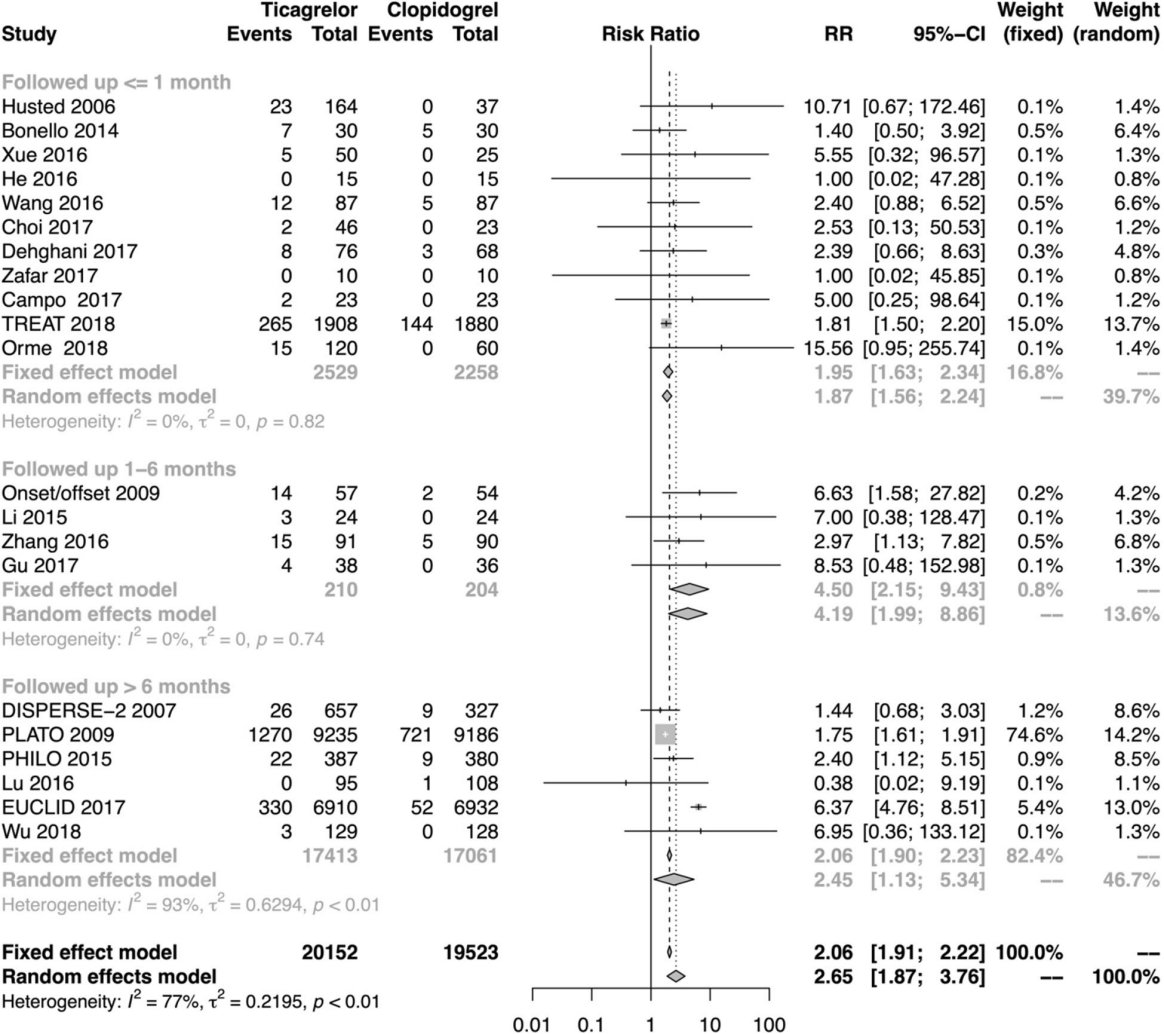
Forest plot of risk ratios for the incidence of dyspnea of ticagrelor vs clopidogrel stratified by follow-up duration

As the increased risk was only found among patients taking ticagrelor, subgroup analysis according to study follow-up duration was only performed in the ticagrelor studies. We found that ticagrelor increased the risk of dyspnea compared with clopidogrel in all follow-up durations, in which the RR was 1.87 (95 CI 1.56–2.24), 4.19 (95% CI 1.99–8.86) and 2.45 (95% CI 1.13–5.34) for follow-up duration less than 1 month, 1–6 months and more than 6 months, respectively. The results are presented in Fig. 3.

### Severity of dyspnea

In the study of Husted 2006 [13], 29 instances of dyspnea were reported by the 23 ticagrelor treated patients, in which 21 were mild and 8 were moderate. But none of them was associated with congestive heart failure or bronchospasm. Three out of fourteen (21.4%) of patients reported dyspnea in the ticagrelor group stopped the study drug owing to dyspnea in the Onset/offset study [15], however, it was only 0.9% in the ticagrelor group and 0.1% in the clopidogrel group in the study of PLATO [2]. It was similar in study of TREAT that few patients discontinued the study drug because of dyspnea (19 of 1913 [1.0%] patients in the ticagrelor group and none in the clopidogrel group). In contrast, dyspnea was one of the most common causes of study drug discontinuation in the study of EUCLID (Examining Use of Ticagrelor in Peripheral Artery Disease) [3].

For patients reported dyspnea in the study of DISPERSE-22007 [14], 27% of the patients had resolution of this symptom within 24 h, 25% after 24 h, and 48% experienced persistent symptoms during treatment (> 15 days). In study of Zhang 2016, there was only 1 patient in whom dyspnea lasted for 1 month, for others dyspnea was tolerable and mild or moderate dyspnoea disappeared within 1 week [23].

### Publication bias

Publication bias were not detected in the overall analysis of dyspnea involving 25 studies (*P* value for Begg’s test: 0.350; *P* value for Egger’s test: 0.246). The funnel plot is presented in Figure S2.

## Discussion

In this study, we performed a meta-analysis using 25 RCTs and found that third-generation oral P2Y_12_ inhibitors have a higher risk of dyspnea than clopidogrel. Compared to clopidogrel, patients taking ticagrelor had twice-fold increase in the risk of dyspnea. In addition, the increased risk of dyspnea for ticagrelor was consistent in subgroup analyses by follow-up duration. However, the adverse effect was not found among patients receiving prasugrel therapy. In addition, ticagrelor induced dyspnea reported in most studies was likely to be mild to moderate or transient, and few patients discontinued ticagrelor because of dyspnea.

The result was similar with the meta-analysis of Caldeira et al., in which they found that ticagrelor, cangrelor, and elinogrel have an increased incidence of dyspnea compared with clopidogrel or prasugrel [35]. But only 5 ticagrelor studies and 1 prasugrel study were included in this study. Tan et al. also assessed the dyspnea risk of ticagrelor and prasugrel as a secondary analysis in a meta-analysis, however, many non-RCTs were included in this meta-analysis [36]. In our meta-analysis, we included 25 studies that reported dyspnea when comparing efficacy or safety of third-generation oral P2Y_12_ inhibitors with clopidogrel, which are all RCTs and decreases the heterogeneity.

A review by Cattaneo and Faioni discussed the dyspnea of new antiplatelet drugs and hypothesized that dyspnea could be related to the reversibility of drug [37]. The study of Caldeira et al. also support this hypothesis that reversible P2Y12 antagonists ticagrelor, cangrelor, and elinogrel have an higher risk of dyspnea in increasing order when compared with irreversible P2Y12 inhibitors such as clopidogrel or prasugrel [35]. This is consistent with the results of our analysis that ticagrelor had a higher risk of dyspnea than clopidogrel, which was not observed in prasugrel.

The mechanism of P2Y_12_ inhibitors, especially ticagrelor, related dyspnea is till be proven. Current hypothesis is inhibition of P2Y_12_ inhibitors, particularly reversible inhibitors, on sensory neurons increasing the sensation of dyspnea [37]. It could be related to the reversibility of drug. Previous also found that dyspnea was mainly found in reversible P2Y12 inhibitors, including cangrelor, elinogrel and ticagrelor [35, 37], which was consistent with our analysis. Another hypothesis is ticagrelor stimulates pulmonary vagal C fibers by inhibiting adenosine reuptake and increasing of extracellular adenosine levels [38]. But there are also evidences against the hypothesis of increased extracellular adenosine by ticagrelor [38].

The DISPERSE-2 trial reported that the increased rate of dyspnea was dose-dependent [14]. But we found that ticagrelor had a higher risk of dyspnea in subgroup studies with standard drug dose, which is commonly used in clinical practice. Though dyspnea was more common in patients using ticagrelor, most of the cases were mild or moderate. A part of patients with dyspnea after taking ticagrelor discontinued the study drug, but the rate varied in different studies. It was only 0.9% in the ticagrelor group in the study of PLATO [2], while it reached 21.4% in the onset/offset study among patients who reported dyspnea [15]. It is consistent in previous studies that this symptom did not last long. Therefore, dyspnea may not be the major concern when using third-generation oral P2Y_12_ inhibitors.

There are several limitations in this study. Firstly, the definition was not specified in the included studies, which may affect the generalization of this study. Second, the follow-up duration ranged among included trials. But we performed a subgroup analysis on ticagrelor stratified by the follow-up duration and we found the result was consistent in studies with different follow-up durations. Third, only four studies on prasugrel were included as most studies did not reported the results of dyspnea. But the current finding on prasugrel was consistent with previous meta-analyses [35, 36].

## Conclusions

In conclusion, a higher risk of dyspnea was found in patients treated with ticagrelor compared with clopidogrel, while it was not observed in patients using prasugrel. Most of the cases, however, were mild or moderate, in spite of a higher risk of dyspnea.

## Supplementary information

**Additional file 1: Table S1.** List of potential bias of included studies. **Figure S1.** Potential bias of included studies using Cochrane Risk Bias. **Figure S2.** Funnel plot of the overall analysis.

## Data Availability

All data generated or analysed during this study are included in this published article.

## Abbreviations

ACS: Acute coronary syndrome
CI: Confidence interval
EUCLID: Examining use of Ticagrelor in peripheral artery disease
PCI: Percutaneous coronary intervention
PLATO: Platelet inhibition and patient outcomes
RCT: Randomized controlled trials
RR: Risk ratio
TRITON-TIMI-38: Trial to assess improvement in therapeutic outcomes by optimizing platelet inhibition with Prasugrel-thrombolysis in myocardial infarction
