# Supplementary material for: The risk of dyspnea in patients treated with third-generation P2Y12 inhibitors compared with clopidogrel: a meta-analysis of randomized controlled trials
Source: BMC Cardiovasc Disord. 2020 Mar 17;20:140. doi: 10.1186/s12872-020-01419-y (PMC7079377; doi:10.1186/s12872-020-01419-y)
Supplement: Supplementary file 1 — Additional file 1: Table S1. List of potential bias of included studies. Figure S1. Potential bias of included studies using Cochrane Risk Bias. Figure S2. Funnel plot of the overall analysis. [file 12872_2020_1419_MOESM1_ESM.docx]

**Supplementary Table 1** List of potential bias of included studies

|  | Selection bias | | Performance bias | Detection bias | Attrition bias | Reporting bias | Selection bias |
| --- | --- | --- | --- | --- | --- | --- | --- |
|  | Random sequence generation | Allocation concealment | Blinding of participants and personnel | Blinding of outcome assessment | Incomplete outcome data | Selective reporting | Other bias |
| Husted (2006) | L | U | L | L | L | L | L |
| DISPERSE-2 (2007) | U | U | L | L | L | L | L |
| PLATO (2009) | U | U | L | L | L | L | L |
| Onset/offset (2009) | L | U | L | L | L | L | L |
| Bonello (2014) | U | U | H | H | L | L | U |
| PHILO (2015) | L | U | L | L | L | L | L |
| Li (2015) | U | U | H | L | L | L | H |
| Zhang (2016) | U | U | H | H | L | L | L |
| Xue (2016) | U | U | L | L | L | L | L |
| He (2016) | L | U | H | L | U | L | L |
| Lu (2016) | U | U | U | U | L | L | H |
| Wang (2016) | U | U | U | U | L | L | U |
| Gu (2017) | U | U | U | U | L | L | U |
| Choi (2017) | U | U | H | H | H | L | L |
| Dehghani (2017) | U | U | H | L | L | L | U |
| EUCLID (2017) | L | U | L | L | L | L | U |
| Zafar (2017) | U | U | H | H | L | L | L |
| Campo (2017) | U | L | H | H | L | L | U |
| TREAT (2018) | L | L | H | L | L | L | U |
| Orme (2018) | U | U | H | H | L | L | L |
| Wu (2018) | U | U | U | U | U | L | U |
| TRITON-TIMI 38 (2007) | U | U | L | L | L | L | L |
| Ge (2010) | U | L | L | L | L | L | L |
| TRILOGY-ACS (2012) | L | U | L | L | L | L | L |
| TRIGGER-PCI (2012) | U | U | L | L | L | L | U |

H, high risk of bias; L, low risk of bias; U, unclear.

**Supplementary Figure 1** Potential bias of included studies using Cochrane Risk Bias Tool

**Supplementary Figure 2** Funnel plot of the overall analysis
